# Letter to editor “A deep learning-based clinical-radiomics model predicting the treatment response of immune checkpoint inhibitors (ICIs)-based conversion therapy in potentially convertible hepatocellular carcinoma patients: a tumor marker prognostic study”

**DOI:** 10.1097/JS9.0000000000003203

**Published:** 2025-08-22

**Authors:** Ben Wang, Naiying Shen, Han Xiao

**Affiliations:** a Department of General Surgery, No. 215 Hospital of Shaanxi Nuclear Industry, Xianyang, China; b Department of Hepato-Biliary-Pancreatic Surgery, The First Hospital of Jiujiang City, Jiujiang, China; c Jiujiang City Key Laboratory of Cell Therapy, The First Hospital of Jiujiang City, Jiujiang, China

*Dear Editor*,

We read with interest the recently published article entitled *“A deep learning-based clinical-radiomics model for predicting durable clinical benefit from immune checkpoint inhibitors in potentially convertible hepatocellular carcinoma patients.”*^[1]^ The authors aimed to integrate radiomics and deep learning approaches with clinical data to predict the efficacy of immune checkpoint inhibitors (ICIs) in patients with potentially convertible hepatocellular carcinoma (HCC). While the study introduces an innovative approach in model development, feature integration, and radiogenomic analysis, several methodological and interpretative issues merit further discussion. We declare that the writing of this article complies with the TITAN guidelines^[2]^.

1. Risk of model overfitting and limited generalizability

This was a single-center retrospective study spanning a relatively long period (2019–2023), during which treatment regimens varied considerably. Although the authors included patients receiving ICIs in combination with anti-angiogenic agents or loco-regional therapies, the individual contributions of these therapeutic modalities to the model’s predictive performance were not clearly addressed. Subgroup analyses, though attempted, were insufficiently detailed to provide robust conclusions significant in the context of increasingly heterogeneous treatment paradigms. Furthermore, the exclusion of patients with poor-quality imaging, missing CT data, or diffuse liver metastases may have introduced selection bias, potentially limiting the generalizability of the model in routine clinical settings.

The study developed both a machine learning model using pyradiomics (XGBoost) and a deep learning model (CNN/ResNet18), later combined into a fusion model via logistic regression. Despite reporting high AUCs (up to 0.96) in both training and test cohorts, the test set included only 50 patients, with 35 showing durable clinical benefit (DCB). Such a limited test sample heightens the risk of overfitting and overestimation of model performance. Ideally, external independent validation or extensive cross-validation would be necessary to assess the robustness and reproducibility of the model.

2. Questionable inclusion of post-treatment AFP response as a predictor

The integration of AFP response defined as a >60% decline post-treatment into the model raises conceptual concerns. As a post-treatment variable, AFP response does not serve as a baseline predictive marker and thus limits the model’s clinical applicability in treatment decision-making. Notably, when AFP response was excluded, model performance declined significantly (AUC decreased to 0.86), indicating that AFP was a major contributor to the model’s predictive capacity. This undermines the intention of developing a purely pre-treatment predictive tool.

3. Limited biological interpretability of radiomic features

The study highlights several key radiomic features (e.g., *wavelet_HHH-glszm_Low Gray Level Zone Emphasis*) using SHAP values, and attempts to relate these to immunogenomic features using TCGA/TCIA datasets. However, the biological interpretation remains relatively superficial. The proposed associations between radiomic features and immune pathways such as MHC-I expression, CD8+ T-cell activity, and TP53 mutations are primarily correlative and lack mechanistic validation. Moreover, potential confounding from prior treatments (e.g., TACE, radiotherapy) on radiomic signatures was not adequately addressed, further complicating the interpretation of radiogenomic links.

In summary, while this study presents a technically advanced framework combining deep learning and radiomics to predict ICIs response in HCC, several limitations persist in study design, model construction, biological interpretability, and clinical utility. Future studies should aim to incorporate larger, multicenter prospective cohorts, distinguish clearly between pre-treatment and post-treatment variables, ensure rigorous model validation, and emphasize clinical deployment strategies. Such efforts will be essential to facilitate the real-world translation of predictive models into personalized immunotherapy for HCC.

## Data Availability

Not applicable.

## Research registration unique identifying number (UIN)

There are no conflicts of interest.
